# The Fast Health Interoperability Resources (FHIR) Standard: Systematic Literature Review of Implementations, Applications, Challenges and Opportunities

**DOI:** 10.2196/21929

**Published:** 2021-07-30

**Authors:** Muhammad Ayaz, Muhammad F Pasha, Mohammed Y Alzahrani, Rahmat Budiarto, Deris Stiawan

**Affiliations:** 1 Malaysia School of Information Technology Monash University Bandar Sunway Malaysia; 2 Information Technology Department College of Computer Science & Information Technology Albaha University Albaha Saudi Arabia; 3 Informatics Department Faculty of Science & Technology Universitas Alazhar Indonesia Jakarta Indonesia; 4 Department of Computer Engineering Faculty of Computer Science Universitas Sriwijaya Palembang Indonesia

**Keywords:** Fast Health Interoperability Resources, FHIR, electronic health record, EHR, clinical document architecture, CDA, Substitutable Medical Applications Reusable Technologies, SMART, HL7, health standard, systematic literature review

## Abstract

**Background:**

Information technology has shifted paper-based documentation in the health care sector into a digital form, in which patient information is transferred electronically from one place to another. However, there remain challenges and issues to resolve in this domain owing to the lack of proper standards, the growth of new technologies (mobile devices, tablets, ubiquitous computing), and health care providers who are reluctant to share patient information. Therefore, a solid systematic literature review was performed to understand the use of this new technology in the health care sector. To the best of our knowledge, there is a lack of comprehensive systematic literature reviews that focus on Fast Health Interoperability Resources (FHIR)-based electronic health records (EHRs). In addition, FHIR is the latest standard, which is in an infancy stage of development. Therefore, this is a hot research topic with great potential for further research in this domain.

**Objective:**

The main aim of this study was to explore and perform a systematic review of the literature related to FHIR, including the challenges, implementation, opportunities, and future FHIR applications.

**Methods:**

In January 2020, we searched articles published from January 2012 to December 2019 via all major digital databases in the field of computer science and health care, including ACM, IEEE Explorer, Springer, Google Scholar, PubMed, and ScienceDirect. We identified 8181 scientific articles published in this field, 80 of which met our inclusion criteria for further consideration.

**Results:**

The selected 80 scientific articles were reviewed systematically, and we identified open questions, challenges, implementation models, used resources, beneficiary applications, data migration approaches, and goals of FHIR.

**Conclusions:**

The literature analysis performed in this systematic review highlights the important role of FHIR in the health care domain in the near future.

## Introduction

### Background

In 2011, the proponent of Australian Health Level Seven (HL7) standards, Grahame Grieve, proposed an interoperability approach called Resources for Healthcare (RFH) as a new standard for better interoperability in digital health. Technically, RFH has been designed for web technology, and the resource is based on extensible markup language (XML) with an HTTP-based representational state transfer (REST)ful protocol and a distinct URL for each resource. The RFH standard was renamed Fast Health Interoperability Resources (FHIR) with extension of previous HL7 specifications (ie, HL7 version 2 and version 3) with consideration of modern web technologies [1].

The main idea behind FHIR was to build a set of resources and develop HTTP-based REST application programming interfaces (APIs) to access and use these resources. FHIR uses components called resources to access and perform operations on patient health data at the granular level. This feature makes FHIR a unique standard from all other standards because it was not available in all previous versions of HL7 (v2, v3) or the HL7 clinical document architecture (CDA).

The basic building blocks of FHIR are the so-called resources, a generic definition of common health care concepts (eg, patient, observation, practitioner, device, condition). FHIR uses JavaScript object notation and XML structures for data exchange and resources serialization. FHIR does not only support a RESTful to exchange resources but also manages and documents an interoperability paradigm.

Since the first day of its introduction, FHIR has gained popularity and has been increasingly adopted by the health care industry. In 2018, six large technology companies, including Microsoft, IBM, Amazon, and Google, pledged to remove barriers for health care interoperability and signed a letter that explicitly mentions FHIR as an emerging standard for the exchange of health data [2]. With incorporation of Substitutable Medical Applications Reusable Technologies (SMART), a platform for interoperable apps [3], FHIR can be expected to attract even more attraction in digital health in the future. Using FHIR for the exchange of medical data can provide potential benefits in a large number of domains, including mobile health apps, electronic health records (EHRs), precision medicine, wearable devices, big data analytics, and clinical decision support.

**Figure 1:**
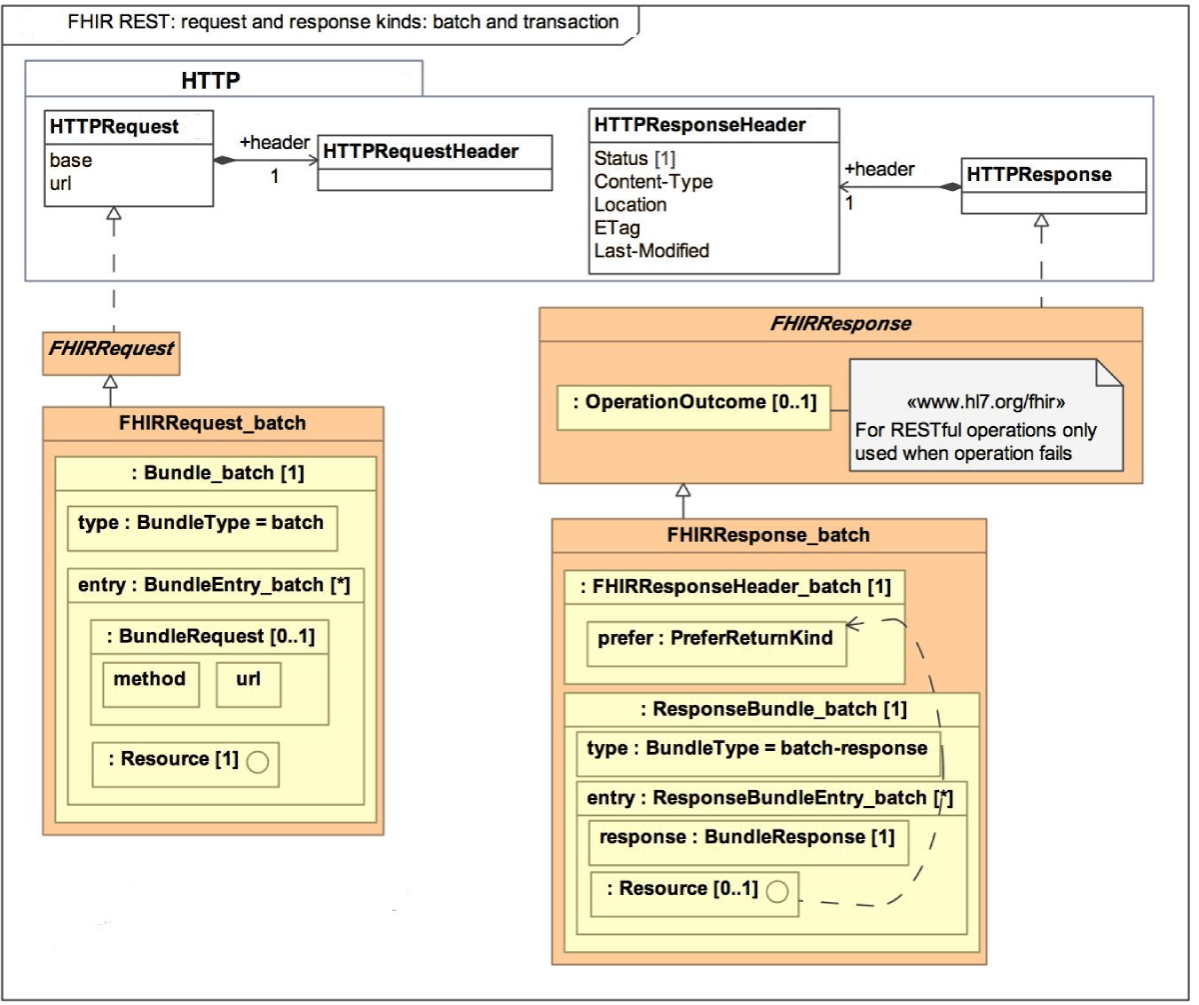
General architecture of the Fast Health Interoperability Resources (FHIR) standard [4].

The main objective of FHIR is to reduce implementation complexity without losing information integrity. Moreover, this new standard combines the advantages of the previous HL7 (v2, v3, and CDA) standards and is expected to overcome their limitations. FHIR allows the developers to develop standardized browser applications that enable the user to access clinical data from any health care system regardless of the operating systems and devices that a health care system uses. For example, a user runs an application on the browser and will access data from a health care system using any device, whether it is running on a desktop, smartphone, Windows, Android, or Linux operating system. Figure 1 represents the general architecture of FHIR [4].

The goal of this study was to gain a deeper understanding of the FHIR standard, and to review the use and adoption of the standard in current health care applications and organizations. This study can assist researchers and experts in understanding the FHIR architecture, design, implementation, resources, challenges, mapping, and adoption in health care informatics. Additionally, this systematic review identifies the key topics discussed in the context of FHIR in the literature.

### FHIR Resource

A resource is the smallest discrete concept that can be maintained independently and is the smallest possible unit of a transaction [5]. Thus, a resource is a known identity providing meaningful data. Each resource has clear boundaries and differs from all other resources. A resource should be described in sufficient detail to define and support the medical data exchange that is involved in the process. According to the latest FHIR version (R4), the FHIR community has defined more than 150 resources to date [6]. These resources are divided into five major categories: (1) Administrative: location, organization, device, patient, group; (2) Clinical: CarePan, diagnostics, medication, allergy, family history; (3) Financial: billing, payment, support; (4) Infrastructure: conformance, document, message profile; and (5) Workflow: encounter, scheduling, order.

FHIR is the latest standard; however, to date, there has been no comprehensive systematic literature review performed in this area. Therefore, a systematic literature review was performed in this study to provide a broad view of FHIR, and to address various challenges, applications, and goals of FHIR highlighted in research in this field.

### Motivation and Objectives

Owing to its dynamic characteristics, FHIR is gaining popularity rapidly. It is expected that FHIR will soon become an icon for clinical information exchange in the health care sector. However, it also faces numerous challenges, which is the main motivation that inspired us to perform this systematic literature review. Despite its importance in health care research, there is no comprehensive review of the literature in the field. 

There were five objectives of this study. The first objective was to profoundly investigate the literature related to FHIR and EHR to explore their multiple challenges in the health care domain and give a comprehensive summary of these issues. The second objective was to identify FHIR applications, goals, challenges, and their roles in the health care domain. The third aim was to address different models of FHIR implementation. Fourth, we addressed different existing and emerging challenges of electronic health implementation to provide the readers with up-to-date information about the different types of hurdles faced by health care project implementations. Finally, this review offers useful suggestions and recommendations about the solutions to these issues faced by health care stakeholders.

## Methods

### Design

This systematic literature review was conducted through the following steps: (1) establishing the research questions to be investigated; (2) identification of digital libraries to be explored and establishing the search strategy; (3) setting the criteria for selection of relevant articles; (4) setting the quality assessment criteria to select the best articles for this study; and (5) data extraction to address the research questions from the selected articles.

### Research Questions

According to Kitchenham et al [7], research questions are the most crucial part of any systematic literature review. Therefore, we have to set questions related to the focus fields, which are FHIR and EHR. We formulated specific research questions to identify the objectives in terms of problems, challenges, solutions, and goals. Our research questions identify the mentioned domain broadly and cover almost every aspect of the field, which is essential for the research purpose. Table 1 summarizes the research questions and their corresponding objectives.

**Table 1:** Research questions and associated objectives.

Research questions	Objectives
SQ1: What are the types or models of FHIR^a^ implementation?	To investigate various techniques, methods, or mechanisms used during the implementation of FHIR
SQ2: What are the common resources used in FHIR implementation?	To identify various resources used during the implementations of FHIR
SQ3: What are the applications that benefit from the use of FHIR?	To identify various types of applications that benefit from the FHIR standard (eg, mobile apps, SMART^b^ on FHIR apps, research apps, HAPI^c^ FHIR apps)
SQ4: What are approaches applied to map or migrate data from previous standards to FHIR?	To investigate various mechanisms on how to extract FHIR resources from HL7^d^ and other previous standards for mapping/migrating to the FHIR standard
SQ5: What are the goals of FHIR?	To identify or investigate the goals of the FHIR standard in the health care domain
SQ6: What are the challenges and open questions related to the FHIR domain?	To explore the challenges in the FHIR domain such as implementations (eg, FHIR API^e^, standard, interoperability)

^a^FHIR: Fast Health Interoperability Resources.

^b^SMART: Substitutable Medical Applications Reusable Technologies.

^c^HAPI: Health Level 7 application programming interface.

^d^HL7: Health Level 7.

^e^API: application programming interface.

### Search Strategy

After establishing the research questions, the next step was to search for articles to collect the required data. To perform a proper systematic literature review, an appropriate search is essential to define the scope and search keywords, which are the fundamental concepts of our research questions for retrieving accurate results.

There is a possibility that the search method may not identify some relevant studies. Therefore, to establish an optimized search string, Kitchenham et al [7] suggest breaking down the research questions into individual facets called research units, which include all of their associated acronyms, synonyms, abbreviations, related words, and alternative spellings combined using Boolean operators (AND, OR) for the construction of keyword phrases.

Finally, we obtained and used the search string shown in Textbox 1 to retrieve the relevant articles.

**Search string for article retrieval.**

[{((Healthcare) or (eHealth) or (EHR)) and ((Standard) or (Protocols))} OR {(FHIR Approaches) or (FHIR Techniques) or (FHIR Methods)} OR {(FHIR) and ((Implementation) or (Challenges) or (Barriers))} OR {(FHIR) and ((Resources) or (HL7 V2) or (HL7 CDA) or (HL7 CDA documents))} OR {(FHIR and SMART) or (SMART on FHIR)} OR {(FHIR) and ((Mapping) or (Exchange))}]

### Article Selection Process

#### Step 1

The following questions were defined for article selection: (1) What are the main domains/fields of the searched papers (eg, FHIR)? (2) Where are these papers published (conferences or journals)? (3) What should be the scope and credibility of these papers? (4) When were the papers published?

#### Step 2

To cover as many studies as possible, we selected the relevant articles from the literature by searching through well-known academic digital databases, including ACM, IEEE Xplore, Springer, Google Scholar, PubMed, and ScienceDirect. These databases cover the most relevant conference and journal articles within the fields of health care and computer science. To limit the search, we set the range from January 2012 to December 2019. The search was performed during January 2020.

#### Step 3

We selected the articles from all of the databases listed above on the basis of the search string (Textbox 1). We used the string and checked every article in chronological order, including title, abstract, keywords, introduction, background, methods, results, discussion, and conclusion. We then selected and downloaded the articles from the databases when the string or substring matched with any string in any of the above components of the article.

#### Step 4

We removed duplicate articles retrieved from different databases, and manually filtered the collected articles using Endnote software to remove the articles included in multiple databases.

#### Step 5

The inclusion criteria were full articles that deal with FHIR published in the English language in world-class conference proceedings or peer-reviewed journals between 2012 and 2019. The exclusion criteria were articles that address an FHIR-related issues but do not meet the inclusion criteria, such as books, theses (doctorate and masters), notes, chapters, press reports, informal literature surveys, literature surveys, papers without access to full text, and articles that discuss aspects outside of the scope of health care without reference to FHIR or EHR. All articles published in non-English journals/proceedings were removed. Table 2 provides further details of the inclusion and exclusion criteria used in this literature survey.

**Table 2:** Inclusion and exclusion criteria.

Criteria	Inclusion	Exclusion
Subject	Full articles that deal with FHIR^a^	Articles that do not deal with FHIR or related acronyms, or do not address issues related to our research questions
Language	Articles published in English journals/proceedings	Articles published in non-English journals/proceedings
Access	Articles that provide access to the full text	Articles without access to the full text
Venue	Articles published in high-impact-factor conference proceedings or peer-reviewed journals	Articles from nonreputable journals/proceedings as well as books, notes, chapters, and press reports
Study type	Primary study	Nonprimary study, including literature review, informal literature surveys, theses, and articles that discuss aspects of health care without reference to FHIR
Publication history	Clear evidence of the article’s print procedure and venue	Publication process with no proper scientific peer review or no clear evidence of the print venue
Keywords	Describe at least one part of our search string	Do not describe any part of the search string

^a^FHIR: Fast Health Interoperability Resources.

## Results

### Characteristics of Selected Articles

After performing the search queries, a total of 8144 articles from all five major digital databases were retrieved from the initial search. After thoroughly checking the web profiles of authors and their networks, 37 new articles were added with a snowballing procedure. From the 8181 retrieved articles, we first applied the duplication criteria, and then set the inclusion and exclusion criteria described in Table 2. Therefore, we first excluded all of the articles found in multiple databases. After removal of duplicates, 1514 articles remained. In the second phase, we discarded articles published in non-English journals/proceedings, resulting in 1442 articles for further screening. In the third phase, we excluded articles that were not primary studies such as reviews and survey papers. Finally, 892 articles remained for further screening.

In the fourth phase, we analyzed the remaining articles on the basis of their title, abstract, and keywords, and the number dropped to 278. In the final phase, after reading and analyzing the full text of the articles, we selected 80 articles from the list to be included in the systematic review. Table 3 shows the results of the different phases of the selection process, Table 4 presents the articles chosen for our study, and Table 5 provides the geographic information of the publications.

As shown in Table 4, the distribution of the articles was 59% and 41% for journal articles and conference proceedings, respectively. The conferences represented are the main international conferences on health care or health care informatics, whereas the journals represent the world-class reputable journals in the field of computer science and health care. In terms of geography, as shown in Table 5, the number of publications related to FHIR published by researchers in the United States was the highest among represented countries. This indicates that the research in the field is quite active in the United States, which may become a factor that pushes the adoption of the standard in the country and in the rest of the world.

The 80 selected articles are arranged based in their primary subject categories in Table 6.

Once the articles were selected, we arranged them by ascending order of publication year. We then considered the attributes of the articles, including author names, article title, venue of publication (eg, journal article, conference proceeding), and publisher name. The complete list of the selected articles and their attributes is depicted in Table 7.

**Table 3:** Phases of article selection and retrieval at each phase.

Phase	Description	Articles included for review, N
1	Total number of articles from all digital databases	8144
2	Snowball sampling	8181
3	Removal of duplicates	1514
4	Exclusion based on language	1442
5	Exclusion based on access and type of study (eg, reviews and survey papers)	892
6	Exclusion based on title, abstract, and keywords	278
7	Exclusion based on full text and nonprimary study	80

**Table 4:** Distribution of article types.

Publication year	Journal articles, N	Conference proceedings, N	Total, N
2012	0	0	0
2013	1	0	1
2014	0	1	1
2015	5	1	6
2016	6	4	10
2017	6	13	19
2018	10	7	17
2019	19	7	26
Total	47 (59%)	33 (41%)	80

**Table 5:** Geographic distribution of the selected articles.

Country	Articles, N	Year
Belgium	2	2018
Canada	4	2018, 2019
Czech Republic	2	2015
France	1	2017
Germany	7	2016, 2018, 2019
Ireland	3	2016, 2018, 2019
Netherlands	5	2016, 2017, 2019
Portugal	3	2017-2019
Switzerland	1	2019
Sweden	1	2017
United Arab Emirates	1	2018
United Kingdom	5	2016, 2017, 2019
United States	45	2013-2019

**Table 6:** Focus of the selected articles over time.

Category	2012, N	2013, N	2014, N	2015, N	2016, N	2017, N	2018, N	2019, N	Total, N
Apps	0	0	1	1	1	6	3	2	14
SMART^a^	0	0	0	0	3	1	1	0	5
FHIR^b^ implementations models	0	0	0	1	1	3	6	1	12
FHIR resources	0	0	0	0	0	2	0	3	5
FHIR framework	0	0	0	0	0	0	0	11	11
Mapping framework/data model	0	1	0	4	4	2	4	4	19
Challenges	0	0	0	0	1	4	3	3	11
FHIR goals	0	0	0	0	0	1	0	2	3
Total	0	1	1	6	10	19	17	26	80

^a^SMART: Substitutable Medical Applications Reusable Technologies.

^b^FHIR: Fast Health Interoperability Resources.

**Table 7:** List of selected articles in ascending order of publication year.

Reference	Title	Year	Publisher	Venue
Bender and Sartipi [8]	HL7 FHIR: an agile and RESTful approach to healthcare information exchange	2013	IEEE^a^	Journal
Lamprinakos et al [9]	Using FHIR to develop a healthcare mobile application	2014	IEEE	Conference
Kasthurirathne et al [10]	Towards standardized patient data exchange: integrating a FHIR based API for the open medical record system	2015	IOS Press	Journal
Franz [11]	Applying FHIR in an integrated health monitoring system	2015	EuroMISE	Journal
Smits et al [12]	A comparison of two detailed clinical model representations: FHIR and CDA	2015	EuroMISE	Journal
Luz et al [13]	Providing full semantic interoperability for the Fast Healthcare Interoperability Resources schemas with resource description framework	2015	IEEE	Conference
Kasthurirathne et al [14]	Enabling better interoperability for healthcare: lessons in developing a standards based application programing interface for electronic medical record systems	2015	Springer	Journal
Jawaid et al [15]	Healthcare data validation and conformance testing approach using rule-based reasoning	2015	Springer	Journal
Rinner and Duftschmid [16]	Bridging the gap between HL7 CDA and HL7 FHIR: A JSON based mapping	2016	IOS Press	Journal
Ulrich et al [17]	Metadata repository for improved data sharing and reuse based on HL7 FHIR	2016	Elsevier	Journal
Ismail et al [18]	HL7 FHIR compliant data access model for maternal health information system	2016	IEEE	Conference
Mercorella et al [19]	An architectural model for extracting FHIR resources from CDA documents	2016	IEEE	Conference
Ruminski et al [20]	The data exchange between smart glasses and healthcare information systems using the HL7 FHIR standard	2016	IEEE	Conference
Doods et al [21]	Converting ODM metadata to FHIR questionnaire resources	2016	Springer	Journal
Bloomfield et al [22]	Opening the Duke electronic health record to apps: Implementing SMART on FHIR	2016	Elsevier	Journal
Lee et al [23]	Implementation of SMART APP Service Using HL7_FHIR	2016	IASER^b^	Journal
Mandel et al [3]	SMART on FHIR: a standards-based, interoperable apps platform for electronic health records	2016	Oxford	Journal
Andersen et al [24]	Point-of-care medical devices and systems interoperability: a mapping of ICE and FHIR	2016	IEEE	Conference
Minutolo et al [25]	Fuzzy on FHIR: a decision support service for healthcare applications	2017	Springer	Conference
Lee et al [26]	Profiling Fast Healthcare Interoperability Resources (FHIR) of family health history based on the clinical element models	2017	Elsevier	Journal
Abbas et al [27]	Mapping FHIR resources to ontology for DDI reasoning	2017	Linköping University	Conference
Yan et al [5]	Clinical decision support Based on FHIR data exchange standard	2017	Atlantis Press	Conference
Diomaiuta et al [28]	A FHIR-based system for the generation and retrieval of clinical documents	2017	Science and Technology Publications	Conference
Subhojeet et al [29]	Attribute based access control for healthcare resources	2017	ACM^c^	Conference
Saleh et al [30]	Using Fast Healthcare Interoperability Resources (FHIR) for the integration of risk minimization systems in hospitals	2017	IOS Press	Journal
Wagholikar et al [31]	SMART-on-FHIR implemented over i2b2	2017	Oxford	Journal
Li and Park [32]	Design and implementation of integration architecture of ISO 11073 DIM with FHIR resources using CoAp	2017	IEEE	Conference
Jiang et al [33]	A consensus-based approach for harmonizing the OHDSI common data model with HL7 FHIR	2017	IOS Press	Journal
Shoumik et al [34]	Scalable micro-service based approach to FHIR server with Golang and No-SQL	2017	IEEE	Conference
Jánki et al [35]	Authorization solution for full stack FHIR HAPI access	2017	IEEE	Conference
Sanchez et al [36]	Achieving RBAC on RESTful APIs for mobile apps using FHIR	2017	IEEE	Conference
Clotet et al [37]	Differentiated synchronization plus FHIR a solution for EMR’s ecosystem	2017	IEEE	Conference
Khalique and Khan [38]	An FHIR-based framework for consolidation of augmented EHR from hospitals for public health analysis	2017	IEEE	Conference
Aliakbarpoor et al [39]	Designing a HL7 compatible personal health record for mobile devices	2017	IEEE	Conference
Hong et al [40]	Shiny FHIR: an integrated framework leveraging Shiny R and HL7 FHIR to empower standards-based clinical data applications	2017	IOS Press	Journal
Leroux et al [41]	Towards achieving semantic interoperability of clinical study data with FHIR	2017	Springer	Journal
Jiang et al [42]	Developing a semantic web-based framework for executing the clinical quality language using FHIR	2017	Elsevier	Conference
Walinjkar and Woods [43]	FHIR tools for healthcare interoperability	2018	Biomedical Research Network	Journal
Kiourtis et al [44]	FHIR Ontology Mapper (FOM): aggregating structural and semantic similarities of ontologies towards their alignment to HL7 FHIR	2018	IEEE	Conference
Jeon et al [45]	Reactive server interface design for real-time data exchange in multiple data source and client	2018	IEEE	Conference
Gopinathan et al [46]	FHIR FLI: an open source platform for storing, sharing and analyzing lifestyle data	2018	Science and Technology Publications	Conference
Lackerbauer et al [47]	A model for implementing an interoperable electronic consent form for medical treatment using HL7 FHIR	2018	Elsevier	Journal
Stan and Miclea [48]	Local EHR management based on FHIR	2018	IEEE	Conference
Ahmad et al [49]	Implementation of SMART on FHIR in developing countries through SFPBRF	2018	ACM	Journal
Walonoski et al [50]	Validation and testing of Fast Healthcare Interoperability Resources standards compliance: data analysis	2018	JMIR	Journal
Urbauer et al [51]	Wearable activity trackers supporting elderly living independently: a standards based approach for data integration to health information systems	2018	ACM	Conference
Kamel and Nagy [52]	Patient-centered radiology with FHIR: an introduction to the use of FHIR to offer radiology a clinically integrated platform	2018	Springer	Journal
Borisov et al [53]	FHIR data model for intelligent multimodal interface	2018	IEEE	Conference
Hussain et al [54]	Learning HL7 FHIR using the HAPI FHIR server and its use in medical imaging with the SIIM dataset	2018	Springer	Journal
Crump et al [55]	Prototype of a standards-based EHR and genetic test reporting tool coupled with HL7-compliant infobuttons	2018	Elsevier	Journal
Peng et al [56]	Linking health web services as resource graph by semantic REST resource tagging	2018	Elsevier	Conference
Sharma and Aggarwal [57]	Mobile based application for predication of diabetes mellitus: FHIR standard	2018	Science Publisher Cooperation	Journal
Alves et al [58]	FHIRbox, a cloud integration system for clinical observations	2018	Elsevier	Journal
Kasparick et al [59]	IEEE 11073 SDC and HL7 FHIR – emerging standards for interoperability of medical system	2018	University of Rostock	Journal
Zohner et al [60]	Challenges and opportunities in changing data structures of clinical document archives from HL7-V2 to FHIR-based archive solutions	2019	IOS Press	Journal
Maxhelaku and Kika [61]	Improving interoperability in healthcare using HL7 FHIR	2019	IDEAS	Conference
Oemig [62]	HL7 version 2.x goes FHIR	2019	IOS Press	Journal
Kiourtis et al [63]	Structurally mapping healthcare data to HL7 FHIR through ontology alignment	2019	Springer	Journal
Metke-Jimenez and Hansen [64]	FHIRCap: transforming REDCap forms into FHIR resources	2019	Elsevier	Journal
Mukhiya et al [65]	A GraphQL approach to healthcare information exchange with HL7 FHIR	2019	Elsevier	Conference
Daumke et al [66]	Clinical text mining on FHIR	2019	Elsevier	Journal
Schleyer et al [67]	Preliminary evaluation of the Chest Pain Dashboard, a FHIR-based approach for integrating health information exchange information directly into the clinical workflow	2019	IOS Press	Journal
Kiourtis et al [68]	A string similarity evaluation for healthcare ontologies alignment to HL7 FHIR resources	2019	Springer	Journal
Kilintzis et al [69]	A sustainable HL7 FHIR based ontology for PHR data	2019	IEEE	Conference
Houta et al [70]	Use of HL7 FHIR to structure data in epilepsy self-management applications	2019	IEEE	Conference
Pfaff et al [71]	Fast Healthcare Interoperability Resources as a meta model to integrate common data models: development of a tool and quantitative validation study	2019	JMIR	Journal
Kondylakis et al [72]	Using XDS and FHIR to support mobile access to EHR information through personal health apps	2019	IEEE	Conference
Hong et al [73]	An interactive visualization tool for HL7 FHIR specification browsing and profiling	2019	Springer	Journal
Semenov et al [74]	Experience in developing an FHIR medical data management platform to provide clinical decision support	2019	MDPI^d^	Journal
Chapman et al [75]	A semi-autonomous approach to connecting proprietary EHR standards to FHIR	2019	Cornell University Library	Journal
Rivera Sánchez et al [76]	A service-based RBAC & MAC approach incorporated into the FHIR standard	2019	Elsevier	Journal
El-Sappagh et al [77]	A mobile health monitoring-and-treatment system based on integration of the SSN sensor ontology and the HL7 FHIR standard	2019	Springer	Journal
Eapen et al [78]	FHIRForm: an open-source framework for the management of electronic forms in healthcare	2019	IOS Press	Journal
Argüello-Casteleiro et al [79]	From SNOMED CT expressions to an FHIR RDF representation: exploring the benefits of an ontology-based approach	2019	RWTH Aachen University	Conference
Jenders [80]	Evaluation of the Fast Healthcare Interoperability Resources (FHIR) standard for representation of knowledge bases encoded in the Arden syntax	2019	Elsevier	Journal
Hong et al [81]	Developing a scalable FHIR-based clinical data normalization pipeline for standardizing and integrating unstructured and structured electronic health record data	2019	Oxford	Journal
Eapen et al [82]	Drishti: A sense-plan-act extension to open mHealth framework using FHIR	2019	ACM	Conference
Mandl et al [83]	Beyond one-off integrations: a commercial, substitutable, reusable, standards-based, electronic health record–connected app	2019	JMIR	Journal
Sharma and Aggarwal [6]	HL-7 based middleware standard for healthcare information system: FHIR	2019	Springer	Journal
Baskaya et al [84]	mHealth4Afrika: implementing HL7 FHIR based interoperability	2019	Elsevier	Journal

^a^IEEE: Institute of Electrical and Electronics Engineers.

^b^IASER: Institute of Applied Social and Economic Research.

^c^ACM: Association for Computing Machinery.

^d^MDPI: Multidisciplinary Digital Publishing Institute.

### Quality Assessment

According to Kitchenham et al [7], it is essential to select and assess the best articles for every literature review, and the quality of selected studies must be verified before inclusion in the study. We evaluated the selected articles with regard to research quality, related work, purposes of research, the obtained result, the methodology used, literature review, current and future objectives, conclusion, publication repository, and other factors. We evaluated the quality of each article according to the protocol defined by Roehrs [85] as displayed in Textbox 2.

**Quality assessment criteria [85].**

- Does the article state the purpose of the research?
- Does the article present the result related to objectives?
- Does the article have a research result?
- Does the article present a literature review and background?
- Does the article present an architecture proposal or research methodology?
- Does the article present a conclusion related to the research objectives?

The proposed quality criteria scores were assessed for each selected article. Although the majority of the selected articles did not fully satisfy all six criteria for evaluation, they complied with at least four out of the six quality assessment criteria listed in Textbox 2.

All of the assessed articles clearly presented their research purpose, literature review, and were supported by a research methodology, bibliographical references, or models/architectural proposals. Based on this quality assessment, we did not exclude any articles from the corpus; this assessment only evaluates whether the articles have a satisfactory structure.

### Data Extraction and Addressing the Research Questions

#### Process

In summary, the quality assessment of the selected articles was as follows. If an article identified by our search query criteria contained information related to our research questions, then the following three steps were applied. First, the title and abstract of the selected articles were carefully read to scrutinize whether the articles were relevant to our research questions. Second, we skimmed the entire article to assure that the required information was available. Finally, in the third round, we read through the entire article from start to end to ensure that this information was helpful for our study and could address the research questions.

To gather information from the selected articles corresponding to our research questions and criteria, we developed separate forms in Microsoft Word and Excel. We reviewed every section of the article from beginning to end and recorded details of the articles in these two forms whenever we found the answer to a corresponding research question. After compilation of the results, we placed these results in specific question-and-answer section tables and discarded the two temporary generated Word and Excel forms.

We collected the following types of data from each article: author name(s), affiliation and country name, venue (journal or conference), publication year. We then collected the answers to the set of research questions from these articles and recorded the details of selected articles for further processing.

#### SQ1: What are the Types or Models of FHIR Implementation?

To address this question, we reviewed the literature in the FHIR domain and investigated various techniques, methods, and mechanisms used in the implementation of FHIR in the health care sector.

At present, FHIR is the most attractive domain among health care researchers. Therefore, extensive efforts are being taken to implement FHIR with consideration of multiple aspects and diverse areas. We obtained 11 categories for FHIR implementation. From a platform point of view, we considered the implementation of mobile/tablet apps [3,9,28,35,39,51,57,60,65,70,72,77,82], standalone apps/servers [3,23,51,58,61], web services/API [3,11,14,26,34,36, 40,48,53,56,71,78], and web-based tools/applications [18,26,37,40,42,46,55,69,78] categories. From a conceptual framework, we considered the categories of general FHIR implementation [48,59], using SMART on FHIR [3,14,22,31], HL7 API (HAPI)-FHIR server/library/applications [14,16,20,32,34-36,40,43,51,54,55,73,76,81,82], and FHIR general framework [14,40,78]. In consideration of compatibility, we chose FHIR data model/data exchange [5,11,26,45,55,66,67,73] and defining ontology to align with FHIR [44,68,69] as the main categories. In addition, we classified all implementation-related work under the miscellaneous category [21,25,30,32,47,60,71], such as FHIR implementation of the legacy clinical data repository system, FHIR implementation of the agent-based system, implementation of operational data model metadata [86] to FHIR questionnaire resource implementation, FHIR implementation of the electronic treatment form, Clinical Asset Mapping Program for FHIR, integration of the architecture of domain information model (ISO/IEEE 11073 DIM) [31] with FHIR, and FHIR-based decision support systems.

#### SQ2: What are the Common Resources Used in FHIR Implementation?

The FHIR community has defined more than 150 resources to date [6]. For this research question, we reviewed the literature in the FHIR domain to identify various FHIR resources used in implementation. We observed that approximately 82 different types of resources have been used in FHIR implementation in various articles. The resource names and the articles mentioned in various resources are shown in Table 8.

In the miscellaneous category, we list all of the articles that mention only one or two resources: (1) Activity Definition, (2) Adverse Reaction, (3) Adverse Event, (4) Address, (5) Billing, (6) Bundle, (7) Contraindication, (8) Conformance, (9) Consent, (10) Concept Map, (11) Claim, (12) Clinical, (13) Clinical Study Plan (14), Clinical Impression (15), Care Team, (16) Category, (17) Coverage, (18) Device Component, (19) Device Observation Report, (20) Document Manifest (21), Document Reference (22), Dosage (23), Data Element (24), Diagnostic (25), Diagnostic Order (26), Drug Administration, (27) Element, (28) Element Definition (29), Equipment (30), Gender (31), Goal (32), Group (33), Intolerance (34), Imaging Study (35), Imaging Manifest (36), Medication Dispense, (37) Message Profile, (38) Nutrition Order, (39) Procedure Request, (40) Provenance (41), Provider, (42) Risk Assessment, (43) Research Definition, (44) Request Group, (45) Relative, (46) Related Person, (47) Schedule, (48) Specimen, (49) Staff (50), Structure Definition.

**Table 8:** List of resources used in Fast Health Interoperability Resources implementation.

Resource name	References
Allergy	[6,10,12,34,49]
Allergy Intolerance	[12,14,19,22,29,33,52,55,58,60,61,72,74,77]
Appointment	[13,55,70]
Condition	[3,10,12,16,18,22,23,26,31,36,40,52,55,60,64,66,71,73,74,76,77,79,81]
Composition	[14,16,70,81]
Care Plan	[6,34,36,39,41,49,61,74,76,77,82]
Device	[6,8,9,11,19,20,24,32,33,39,43,48,53,77]
Device Metric	[24,32,53]
Detected Issue	[25,74,77]
Document	[6,8,33]
Diagnostic Report	[28,44,52,60,63,74,84]
Encounter	[10,16,21,30,33,39,41,55,71,74,77]
Episode Of Care	[21,41,77,84]
Family Member History	[22,26,27,60,61,74,77,81]
Family History	[6,22,33]
Immunization	[22,60,74]
Location	[6,10,14,16,24,34,49,71,77]
Medication	[3,9,16,19,27,29,31,33,39,40,48,49,70,77,81]
Medication Administration	[60,70,71]
Medication Order	[16,22,23,70]
Medication Statement	[19,27,55,66,77,81]
Medication Prescription	[3,22,31]
Medication Request	[66,71,73,74,77]
Observation	[3,9-11,14,18-26,28-33,36,39-41,43,44,48,49,51-53,55,58,60,63,64,66,69-71,73-77,79,82,84]
Organization	[5,6,16,19,34,39,41,75,77,84]
Patient	[3,5,6,8-10,12,14,16-24,27-32,34,36,39-41,44,48-50,52,53,55,58,61,63,65,68,70-77,84]
Person	[14,46,69]
Plan Definition	[41,74,80]
Practitioner	[9,17-19,24,27-29,34,41,44,48,52,53,58,61,63,71,77]
Procedure	[3,40,60,66,71,72,74,77,81]
Questionnaire Response	[21,41,47,60,69,70,74,78,84]
Questionnaire	[17,21,41,47,64,65,70,74,84]
Miscellaneous	[3,5,6,10-12,14-17,21,23,24,28,29,32-34,41,44,46,47,49,52,55,60,66,71-75,77]

#### SQ3: What are the Applications that Benefit from the use of FHIR?

We attempted to thoroughly investigate the literature from various directions and provide the readers with a comprehensive summary of every aspect of FHIR. In this section, we consider the type of applications that can benefit from the FHIR standard, including health care systems/applications benefit in terms of interoperability/data exchange, rules, security/privacy, conformance, health care process, and administration. Thus, we came up with eight categories based on how the applications make use of the FHIR standard (Table 9). In the miscellaneous category, we included all articles that address the type of applications that benefit from the FHIR standard but do not fall under any of the other categories mentioned above (eg, clinical applications for data exchange, testing applications). Table 9 shows the articles that address specific applications that benefit from the FHIR standard.

**Table 9:** Applications that benefit from the use of Fast Health Interoperability Resources (FHIR).

Applications types	References
Mobile apps	[5,9,25,32,34,35,39,40,42,49,57,59,67,72,73,76,77,82]
SMART^a^ on FHIR	[3,14,22,49,67,72,73,83]
Research	[5,15,18,25,40,42,55,60,64,66,67,73,78]
Electronic records and medical practices	[25,39,46,52,55,67,73]
HAPI^b^ FHIR	[14,26,34,40,43,73,81]
Graphic/images	[8,52,61,67]
Web-based	[34,42,55,59,67,73]
Miscellaneous	[26,50,60]

^a^SMART: Substitutable medical applications reusable technologies.

^b^HAPI: Health Level 7 application programming interface.

SMART on FHIR is mentioned under implementation in Table 8 as well as under applications in Table 9. Articles that mentioned SMART on FHIR implementation, either fully or partially, were grouped into one category, and the articles that mentioned any applications that benefit from the SMART on FHIR concept were considered as a different category. Thus, in Table 8, we list articles that mention SMART on FHIR in various implementations, whereas in Table 9, we list applications that benefit from SMART on FHIR platforms.

The SMART platform is a health data layer based on the FHIR API and resource definitions [87]. From the beginning, the SMART team selected platform components that emphasize web standards (eg, HTML, JavaScript, OAuth, and Resource Description Framework) [3]. This setup results in the HL7 legacy versions (ie, v2, v3, CDA) to be unable to implement SMART applications. All previous versions could not use a web API for data access and were unable to access data at the granular level. Additionally, the CDA is based on the reference information model and is lacking in sufficient detail, whereas version 2 suffers from inconsistencies across implementations and version 3 is complex, which leads to incompatible documents and systems [3].

In contrast, the FHIR standard uses web APIs for data access, which is capable of accessing the clinical data at the granular level. The SMART on FHIR concept does not exist without support of the FHIR standard. Therefore, the SMART concept is developed after introducing the FHIR standard, and SMART on FHIR when considered as a standard has some predecessors. All of these contribute in one way or another to the current standards (FHIR) [88]. Considering all of this evidence, we conclude that SMART on FHIR is the main beneficiary of the FHIR standard compared with the other standards.

During the literature review, we observed that mobile, research, and SMART on FHIR applications are the most common beneficiaries of the FHIR standard, followed by electronic records and medical practices, and web-based applications.

#### SQ4: What Approaches are Applied to Map or Migrate Data from Other HL7-Based Legacy Systems to the FHIR-Based System?

At present, HL7 (v2 and CDA) is the most popular data standard in the health care sector, with many countries still using this standard for medical data exchange. Specifically, more than 35 countries implement the HL7 v2 standard and 95% of US health care organizations are still using this standard for medical data sharing among various health care organizations [89].

Owing to its dynamic structure, FHIR provides numerous advantages such as flexibility to manage and retrieve granular clinical information from the whole document. Clinical practitioners and health care providers expect that the FHIR standard will soon occupy the health care market, and that it will replace all of the previous HL7 (eg, v2, v3, CDA) standards. Nevertheless, in this review, we found that FHIR is not likely to replace the previous HL7 (v2, v3, and CDA) standards within weeks or months, but might take years or decades. The rationale is related to the worldwide implementation of the earlier standards such as HL7 v2 and HL7 CDA. Furthermore, health care organizations argue that FHIR has not yet replaced the ubiquitous HL7 v2, and likely will not for several years, because many organizations have already recognized the value of adopting FHIR alongside legacy HL7 standards [27].

Therefore, for addressing this research question, we reviewed the literature in the FHIR domain and investigated various articles that address the mechanisms to extract FHIR resources from HL7 or other previous standards, and to map or migrate them to the FHIR standard. We classified these mappings into six different categories (Table 10).

All of the included articles that address the mapping of any standards to the FHIR standard, but that are not in the six categories mentioned above, were categorized as “miscellaneous/other standards to FHIR mapping.” Nevertheless, we found one study in which data were mapped from the FHIR standard to other standards, and the FHIR resource was mapped to the Web Ontology Language–based ontology [27]. Table 10 shows the list of articles categorized into different mapping categories.

**Table 10:** Approaches used to map or migrate data from other Health Level 7 (HL7)-based legacy systems to the Fast Healthcare Interoperability Resources (FHIR)-based system.

Techniques or methods	References
Map HL7 version 2 to FHIR	No relevant articles
Map HL7 CDA^a^ documents, C-CDA^b^, or HL7 version 3 to FHIR	[5,8,12,16,19,60,79]
ODM^c^ to FHIR	[21,41,64]
Map FHIR to other	[27]
i2b2^d^ to FHIR format	[31,71]
Health record data model to FHIR	[38,63,68,75,81,84]
Map other standards to FHIR	[13,17,24,33,64,71]

^a^CDA: clinical document architecture.

^b^C-CDA: consolidated clinical document architecture.

^b^ODM: operational data model.

^c^i2b2: Informatics for Integrating Biology and the Bedside.

#### SQ5: What are the Goals of FHIR?

For this research question, we reviewed the literature in the FHIR domain to identify or investigate the goals of the FHIR standard in the health care domain. According to the objectives of the reviewed articles, we divided the inquiries regarding the goals into seven different objectives (Table 11). Table 11 shows the articles that address various goals of the FHIR standard, demonstrating that most of these articles focus on the result rather than other goals and objectives.

**Table 11:** Goals of Fast Healthcare Interoperability Resources.

Goals	References
Simplify implementation without sacrificing information integrity	[5]
Patient satisfaction	[73]
Solve health problems (administrative and clinical)	[25,49,52]
Improve global health data interoperability	[49,59,67]
Enhance and maintain quality of data and accessibility	[60]
Result	[15,18,19,27,52,55,73]

#### SQ6: What are the Challenges and Open Questions Related to FHIR?

As the latest standard in the health care domain, it is predictable that FHIR will face various challenges in terms of implementation, adoption, maintenance, data exchange, and other issues. In addition, numerous questions will be raised with respect to use of the FHIR standard. Therefore, for this research question, we reviewed the literature in the FHIR domain to identify various challenges faced by the FHIR standard. We found 19 articles that discussed the implementation challenges, highlighting seven areas of challenge for the FHIR standard (Table 12).

Observations made during the literature review led us to conclude that implementing FHIR in any type of application is the most challenging task in the health care sector; 9 of the 19 related articles discussed this issue. Developers face various types of challenges during the development of any FHIR-based application. Table 12 lists the articles that mentioned these challenges.

**Table 12:** Challenges and open questions related to Fast Healthcare Interoperability Resources (FHIR).

Challenges	References
Implementations of FHIR in an application	[8,12,40,42,48,49,62,64,77]
Standards complexity	[8,27,62,81]
Adoptions	[40,56,83]
FHIR maintenance and specification	[41,42,62]
REST^a^ful approach	[56,59,65,73]
Mapping/migration challenging	[71,75,81]
Miscellaneous	[12,49]

^a^REST: representational state transfer.

## Discussion

### Principal Findings

This systematic literature review successfully identified both qualitative and quantitative sets of studies that enable obtaining a clear view of the FHIR standard in health care in the past 8 years, starting from the selected number of articles. Some of the most relevant studies in the field are highlighted according to systematic selection criteria. We identified the main topics associated with the use of FHIR in digital health. Many articles dealt with topics related to FHIR implementations and use resources, as well as data migration data models. As expected, various application categories such as mobile apps, SMART on FHIR applications, and research applications were the main topics associated with FHIR. Multiple challenges in FHIR adoption and implementation were also highlighted in the included articles. Interestingly, only a small number of relevant articles addressed FHIR goals.

At the beginning of this study, we planned to identify some common aspects in this field by answering some fundamental research questions. Hence, we established six research questions to address the objectives, goals, applications, and challenges of FHIR emerging in recent years. As a result, we can propose a taxonomy of the literature, and identify gaps to be further investigated on existing challenges and issues related to use of the FHIR standard in recent years. We also identify other common and related aspects with respect to interoperability, privacy, authorization (access control), data type, and testing and validation tools. For example, interoperability between the provider and hospital systems poses additional barriers to effective data sharing. In addition, various testing and validation tools are used to improve server compliance with the FHIR specification. Moreover, FHIR specifications define different data types to access and process the FHIR resources element.

Various FHIR-related studies aim to address FHIR implementation challenges such as data migration and cross-institutional sharing of clinical data in the clinical environment [60,71]. The main findings that are presented in these reviews and some other related studies include the importance of realizing EHR data interoperability via adoption of FHIR by health care providers. This adoption might be essential for the improvement of health care services with respect to health data sharing, integration, and availability. Furthermore, use of the FHIR standard in the health care sector may enhance the chance of adoption of smart technologies in the health care domain, such as smartphones, mobile health apps, tablets, smart watches, fitness trackers, and any other future innovations [90]. Furthermore, use of artificial intelligence technologies and data sciences will also be dominant in implementing FHIR-based applications.

FHIR is viewed as the latest standard purely operating on resources, which are used for data storage, migration, and processing among multiple health care providers. The resources-based structure of FHIR is declared as distinct from other standards and is considered to be its main advantage. FHIR has several advantages that range from being a flexible standard, minimal implementation complexity, ability to display the patient history in a single document, granular data access, and avoiding message variability with the use of RESTful APIs.

FHIR is considered to be a unique pathway that can offer a solution to the interoperability issues of clinical data. Nevertheless, various studies indicate that FHIR also faces numerous challenges such as implementation, adoption, maintenance, mapping, and standard complexity. The RESTful API that makes FHIR unique from other standards also faces its own challenges in accessing sensitive health care data stored in the cloud environment [36].

Numerous studies have shown that several applications used in different domains are taking advantage of FHIR, including mobile apps, SMART on FHIR applications, research applications, electronic records and medical applications, graphic/image applications, HAPI FHIR application, and web-based applications.

To the best of our knowledge, this is the first comprehensive systematic literature review that focuses on FHIR-based EHR. There are some systematic literature reviews available in the FHIR domain; however, we found that the existing reviews are unable to explain the FHIR standard in detail. The FHIR standard is very rich, and therefore research in this domain is equally diverse with focus in various directions. Readers are interested in searching for articles that do not only introduce the FHIR standard but also explain various aspects of the standard in detail. For example, Lehne et al [91] only reviewed articles related to a general introduction of FHIR, without providing in-depth analysis on either FHIR or articles mentioning FHIR. In particular, the titles or identification of included articles and more comprehensive details of the included articles are missing. Based on a thorough reading, we concluded that this previous review could not fully introduce and address various aspects of FHIR, such as challenges, applications, goals, mapping, and implementation models. Moreover, individual aspects of the reviewed articles were not explained adequately. Therefore, it is not possible to find a corresponding article when interested in a particular topic. Further, the authors focused on articles published between 2002 and 2018, although the FHIR concept was only introduced in 2011; thus, this was a mixed review of EHR and FHIR with little focus on FHIR itself. Lastly, the authors included only 15 references in the review, which is not sufficient for a systematic literature survey.

Similarly, another systematic literature review [90] only explained the general concept and current status of FHIR, whereas core issues such as challenges, goals, and application implementation were not discussed. Important articles that discuss the FHIR resources used in various application implementations were also not included in this review. Although this previous review analyzed some articles in the FHIR domain, the list of articles was not provided or explained properly. Therefore, it is quite difficult for readers to search for articles related to specific information of interest, such as FHIR applications, goals, challenges, and used resources. This information is the core requirement for readers interested in this field. Thus, we concluded that the existing reviews only introduced the FHIR standard without performing a comprehensive analysis of the current state of the field.

In this work, we deeply explored the literature and identified articles that not only mention the FHIR standard but also discuss its major aspects such as core challenges, applications, goals, mapping, used resources, and implementation models. In addition, we highlighted every article along with their references and addressed aspects such as those mentioned above. We searched existing databases for articles on the FHIR standard published between 2012 and 2019, and then included every article that discussed or mentioned even a single aspect of the FHIR standard. This approach provides a convenient resource for readers to easily search articles of interest in the literature.

FHIR is a new standard in the health care domain. It is still in the early stages of development and evaluation, and consequently faces numerous obstacles. We believe that these obstacles might eventually be overcome, thereby opening a new roadmap to solving the problem of data interoperability in the health care sector, which is in line with the findings of the literature review and remains the main objective of our research.

### Limitations

This study was limited to aspects related only to the FHIR standard rather than the general health care concept. In this sense, the literature review focused exclusively on articles addressing FHIR concepts. This work sought to answer research questions proposed for providing an outline of the current literature related to FHIR without specifically assessing any computer system that refers to FHIR use. In addition, our search focused on articles published in various scientific journals related to health care and computer science within a limited time frame. This investigation was limited to articles selected from journals/conferences through implementations of standard steps of the systematic literature review methodology. We focused on scientific articles and did not address commercial or more technological approaches or solutions.

### Conclusion

This study provides a systematic literature review regarding the FHIR EHR standard, with the main objective of identifying and discussing the main issues, challenges, goals, and possible benefits from adoption of the FHIR standard in the health care sector. We have explored the FHIR-related literature and investigated articles associated with the FHIR standard in health care information systems. We identified various data models, methods/techniques used in FHIR implementation, FHIR beneficiary applications, and resources used in FHIR implementation. Various data mapping techniques/approaches, key challenges, and primary goals of FHIR were also explored. We observed that FHIR studies mainly focus on clinical data interoperability and portability issues between health care information systems.

The FHIR standard is capable of providing an optimized solution for medical data exchange between two systems and will establish data-sharing trust among health care providers. Furthermore, the FIHR standard is identical in terms of the support of smart technologies such as smartphones, tablets, mobile health apps, smart watches, and fitness trackers, which could solve numerous health care problems that were not possible for the previous standards (ie, HL7 v2, v3 and CDA). Based on this thorough investigation of the literature, we recommend the FHIR standard as a future suitable solution for addressing the health care interoperability problem. Nevertheless, FHIR itself faces some challenges such as implementation, standard complexity, and adoption, among others. Therefore, further research is required to address these challenges.

This review on the standard, purpose, and applications of FHIR will provide readers with a more comprehensive view and understanding of FHIR. This review should also help researchers and health care information technology professionals to access FHIR-associated information in the research community and to assess its impact on digital health. Lastly, this work can provide a roadmap, and suggest possible directions for future research and development in the FHIR domain.

## Abbreviations

API: application programming interface
CDA: clinical document architecture
EHR: electronic health record
FHIR: Fast Healthcare Interoperability Resources
HAPI: Health Level 7 application programming interface
HL7: Health Level 7
REST: representational state transfer
RFH: Resources for Healthcare
SMART: substitutable medical applications reusable technologies
XML: extensible markup language
