# Correction: The Fast Health Interoperability Resources (FHIR) Standard: Systematic Literature Review of Implementations, Applications, Challenges and Opportunities

**DOI:** 10.2196/32869

**Published:** 2021-08-17

**Authors:** Muhammad Ayaz, Muhammad F Pasha, Mohammed Y Alzahrani, Rahmat Budiarto, Deris Stiawan

**Affiliations:** 1 Malaysia School of Information Technology Monash University Bandar Sunway Malaysia; 2 Information Technology Department College of Computer Science & Information Technology Albaha University Albaha Saudi Arabia; 3 Informatics Department Faculty of Science & Technology Universitas Alazhar Indonesia Jakarta Indonesia; 4 Department of Computer Engineering Faculty of Computer Science Universitas Sriwijaya Palembang Indonesia

In “The Fast Health Interoperability Resources (FHIR) Standard: Systematic Literature Review of Implementations, Applications, Challenges and Opportunities” (JMIR Med Inform 2021;9(7):e21929) the authors noted one error.

The title of the originally published article contained an error in the abbreviation “FHIR.” The title originally read as follows:

The Fast Health Interoperability Resources (FIHR) Standard: Systematic Literature Review of Implementations, Applications, Challenges and Opportunities

In the corrected version, the title has been revised to:

The Fast Health Interoperability Resources (FHIR) Standard: Systematic Literature Review of Implementations, Applications, Challenges and Opportunities

The correction will appear in the online version of the paper on the JMIR Publications website on August 17, 2021, together with the publication of this correction notice. Because this was made after submission to PubMed, PubMed Central, and other full-text repositories, the corrected article has also been resubmitted to those repositories.

